# The multidrug-resistant *Pseudomonas fluorescens* strain: a hidden threat in boar semen preservation

**DOI:** 10.3389/fmicb.2023.1279630

**Published:** 2023-10-06

**Authors:** Zhixuan Xiong, Ziqiang Hong, Xinxin Li, Dongyang Gao, Linkang Wang, Shudan Liu, Junna Zhao, Xiangmin Li, Ping Qian

**Affiliations:** ^1^National Key Laboratory of Agricultural Microbiology, Hubei Hongshan Laboratory, Huazhong Agricultural University, Wuhan, Hubei, China; ^2^College of Veterinary Medicine, Huazhong Agricultural University, Wuhan, Hubei, China; ^3^Key Laboratory of Preventive Veterinary Medicine in Hubei Province, The Cooperative Innovation Center for Sustainable Pig Production, Wuhan, Hubei, China

**Keywords:** boar semen preservation, *Pseudomonas fluorescens*, microbial resistance, whole-genome sequencing, antibiotic resistance island

## Abstract

Although the bacterial composition of boar ejaculate has been extensively studied, the bacterial composition of extended boar semen is often overlooked, despite the potential risks these microorganisms may pose to the long-term preservation of extended boar semen at 15–17°C. In this study, we characterized the bacterial community composition of extended semen and discovered that *Pseudomonas* spp. was the dominant flora. The dominant strains were further isolated and identified as a potential new species in the *Pseudomonas fluorescens* group and named *GXZC* strain, which had adverse effects on sperm quality and was better adapted to growth at 17°C. Antimicrobial susceptibility testing showed that the *GXZC* strain was resistant to all commonly used veterinary antibiotics. Whole-genome sequencing (WGS) and genome annotation revealed the large genetic structure and function [7,253,751 base pairs and 6,790 coding sequences (CDSs)]. Comparative genomic analysis with the closest type strains showed that the *GXZC* strain predicted more diversity of intrinsic and acquired resistance genes to multi-antimicrobial agents. Taken together, our study highlights a problem associated with the long-term storage of extended boar semen caused by a *P. fluorescens* group strain with unique biological characteristics. It is essential to develop a new antibacterial solution for the long-term preservation of boar semen.

## Introduction

Artificial insemination (AI) is widely used in global pig production to facilitate improvements in fertility, genetics, labor, and herd health (Knox, 2016). In pig AI, more than 99% of boar semen is stored in a nutrient-rich liquid state at 15–20°C (Johnson et al., 2000; Pezo et al., 2019). Long-term extenders are now widely used in commercial extended semen to meet the rapid development of AI and increase the flexibility of semen use, based on their ability to preserve sperm for 7–12 days after collection (Karageorgiou et al., 2016). However, the boar semen collection process is not aseptic, and freshly collected boar ejaculate often contains bacterial contamination which sources animal (e.g., feces, hair, and human) and non-animal (e.g., water, feed, and air) (Althouse et al., 2000). The bacterial composition of boar ejaculate is complex (Althouse et al., 2000, 2008; Althouse and Lu, 2005; Godia et al., 2020; Zhang et al., 2020). Some bacterial strains isolated from boar semen have been shown to reduce sperm quality (motility, plasma membrane integrity, acrosome, etc.), such as Enterobacteriaceae family (Luis Ubeda et al., 2013), *Clostridium perfringens* (Sepulveda et al., 2013), *Pseudomonas aeruginosa* (Sepulveda et al., 2016), *Enterobacter cloacae* (Prieto-Martinez et al., 2014), *Staphylococcus aureus* (Li et al., 2017), *Proteus mirabilis* (Gao et al., 2018), and *Proteus vulgaris* (Delgado-Bermudez et al., 2020). Previous studies have reported bacterial thresholds between × 10^3^ and × 10^7^ CFU/ml before adverse effects on sperm quality or fertility become apparent (Auroux et al., 1991; Diemer et al., 1996; Bussalleu et al., 2011; Sepulveda et al., 2013, 2014; Prieto-Martinez et al., 2014; Pinart et al., 2017; Delgado-Bermudez et al., 2020).

Antibiotics are commonly added to commercial extenders as the primary approach to control bacteria (Althouse et al., 2000). According to national regulatory requirements (Union, 1992; CSAMR, 2019), antibacterial substances, as essential components of commercial extenders, are strictly regulated by the requirements of local laws or regulations. Antibiotics commonly used in semen diluents include the β-lactams (penicillins, cephalosporins), aminoglycosides (gentamicin, streptomycin, and amikacin), macrolides (tylosin, spectinomycin), and lincosamides (lincomycin) (Santos and Silva, 2020). Aminoglycosides are the most commonly used antibiotics in boar semen (Mazurova and Vinter, 1991; Bryla and Trzcinska, 2015; Waberski et al., 2019).

Recently, the demand for long-term semen preservation has increased in the efficient and competitive pig industry in China. However, long-term storage in nutrient-rich container with antimicrobial agents facilitates the development of drug resistance in bacteria. The potential risks associated with bacterial contamination in long-term storage of extended boar semen cannot be ignored. Therefore, this study aimed to characterize the composition of the extended boar semen microbiome, isolate the predominant microorganisms, and explore their potential risks.

## Materials and methods

### Sampling processing

**Figure 1:**
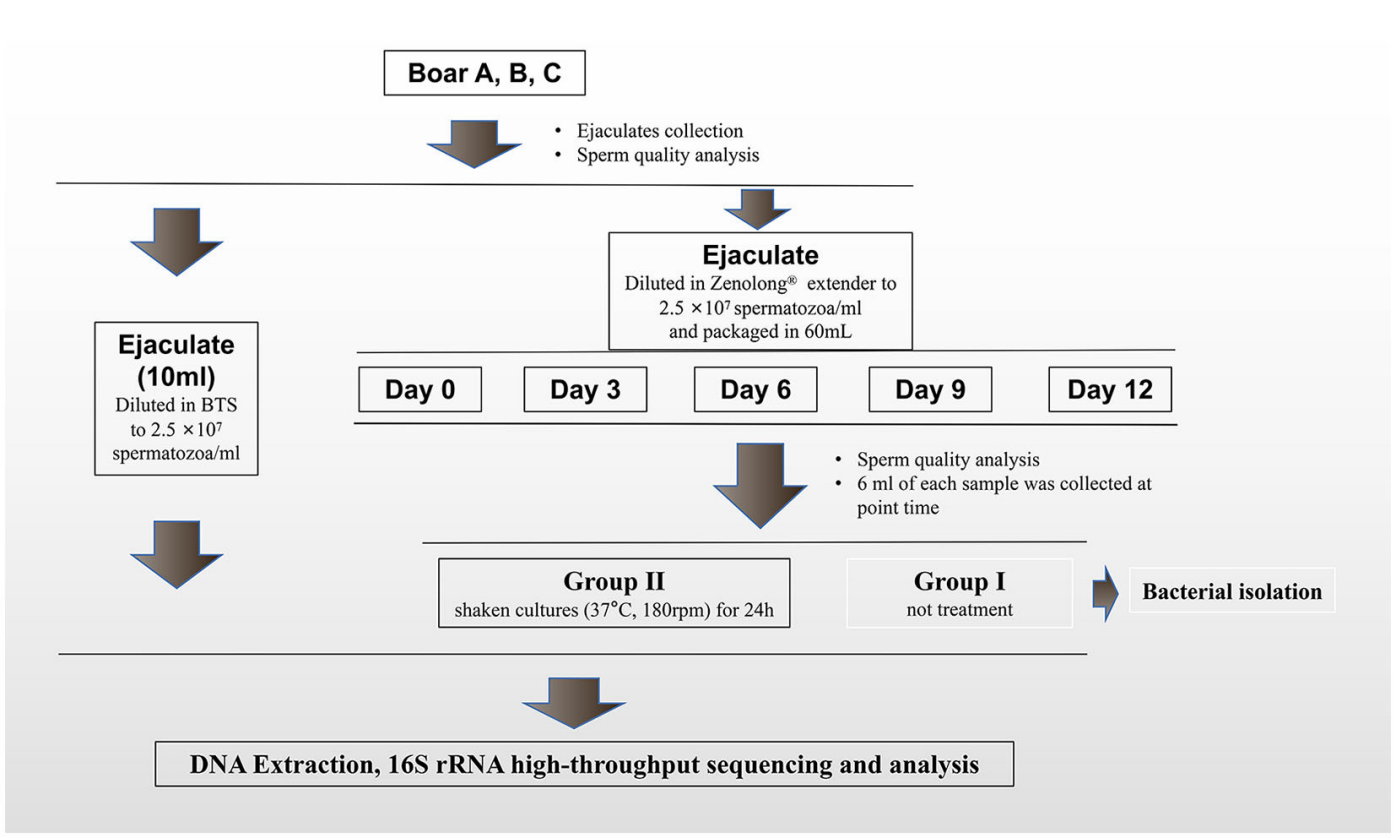
Sampling processing.

Three healthy and sexually mature (1–2 years of age) boars were randomly selected from the artificial insemination (AI) station of Yangxiang Farming Co., Ltd., which is a floor building with air filtration, temperature, humidity, and wind speed-controlled automatically. Boars were farmed in the same condition. The brief procedures are presented in Figure 1.

The collection and processing of ejaculates were conducted according to the minimum bacterial contamination protocol (Althouse et al., 2000). Each ejaculate was filtered through gauze to remove the gel. In total, 10 ml of ejaculate is diluted in sterile Beltsville thawing solution (BTS) to the concentration of 2.5 × 10^7^ spermatozoa/ml (Pursel and Johnson, 1975) and seems as antibiotic-free group (detailed descriptions in the Supplementary material). Each of the remaining ejaculates was processed with the commercial manufacturing requirements of the AI station. In brief, the ejaculates diluted in Zenolong^®^ (Beikang, Taizhou, China; with gentamicin added), to the same sperm concentration, and cooled to 17°C. Then, they were packaged in 60 ml plastic bags (IMV, Shanghai, China). Three of packaged extended semen doses of each boar were stored at 17°C for 12 days. After 0, 3, 6, 9, and 12 days of fluid storage at 17°C, the extended semen total sperm motility (TSM, including progressive motility and non-progressive motility) was measured using a computer-assisted semen analysis (CASA) system (HTR-IVOS II, Hamilton Thorne Research, Beverly, MA, United States; software settings are presented in Supplementary Table S1), and composition of bacterial community was characterized by 16S rRNA high-throughput sequencing. Considering the low bacterial concentrations which were not 16S rRNA sequenced for analysis at the early stages of stored extended semen, samples collected each time were shaken (37°C, 180 rpm) for 24 h to get the high-concentration bacterial communities and seem as group II. Previously unprocessed samples were seemed as group I.

### DNA extraction and 16s rRNA high-throughput sequencing

The MagBeads Fast DNA^TM^ Kit for Soil (MP Biomedicals, Santa Ana, CA, United States) was used to extract total genomic DNA from groups I and II. The concentration was determined using a fluorometer (Qubit Fluorometer, Invitrogen), and <0.3 ng/μl did not meet the detection requirements. The samples were discarded.

PCR amplification of the bacterial 16S rRNA gene V3-V4 region was performed using the forward primer 338F and reverse primer 806R (Chen et al., 2017; Hu et al., 2018). The PCR products were purified using the QIAquick PCR Purification Kit (Qiagen, China), followed by end-repair mix and incubation at 20°C for 30 min. The end-repaired DNA was, then, purified, followed by A-tail mix and incubation at 37°C for 30 min. The purified adenylated 3′-end DNA was combined with adapter and ligation mix, and the ligation reaction was incubated at 16°C for 12–16 h. Adapter-ligated DNA was selected by running a 2.5% agarose gel for approximately 2.5 to 3 h to recover the target fragments. The gel is purified using QIAquick Gel Extraction Kit (Qiagen, China). The final library was quantified using two methods: determining the average molecular length using the Agilent 2100 Bioanalyzer instrument and quantifying the library by real-time quantitative PCR (qPCR) (TaqMan Probe). The raw reads were filtered to remove adaptors and low-quality and ambiguous bases. The paired-end reads were, then, merged to the tags using the Fast Length Adjustment of SHort reads (FLASH) (version 1.2.11, http://ccb.jhu.edu/software/FLASH/) (Magoc and Salzberg, 2011). These tags were clustered into operational taxonomic units (OTUs) at a 97% cutoff value using UPARSE software (version 7.0.1090, http://drive5.com/uparse/) (Edgar, 2013). Chimera sequences were detected by comparing the Gold database using UCHIME (version 4.2.40, http://drive5.com/uchime/uchime) (Edgar et al., 2011). The OTU representative sequences were, then, taxonomically classified with a minimum confidence threshold of 0.6 using the Ribosomal Database Project (RDP) Classifier (version 2.2, http://rdp.cme.msu.edu/) and trained on the Greengenes database (version 201305) using QIIME (version 1.8, http://qiime.sourceforge.net/) (Caporaso et al., 2010). The OTU abundance statistics table of each sample was obtained by comparing all tags with the OTUs using the USEARCH global method (version 7.0.1090, http://www.drive5.com/usearch) (Edgar, 2010). Bar graphs of different classification levels were plotted using the R package (version 3.4.1).

### Bacterial isolation and characterization

The samples stored for the last day of group I were inoculated on TSA and stored at 17°C and 37°C, respectively, for 36 h. Five colonies with visually distinguishable colony morphologies were picked from the 17°C and 37°C samples. These colonies were designated as GXZC-number. The single primary colony was continuously purified for three passages. The resulting colonies were subjected to 16S DNA sequencing (Tsingke, Wuhan, China), and the sequences were identified using the BLAST (blastn) tool on the National Center for Biotechnology Information (NCBI) website (https://www.ncbi.nlm.nih.gov/).

The growth of the isolated strains was analyzed in flat-bottomed 100-well microtiter plates by measuring the optical density at 600 nm (OD600) every 30 min using the Bioscreen C system (Labsystems Oy, Helsinki, Finland) with shaking (180 rpm) at 17°C, 27°C, and 37°C.

Antimicrobial susceptibility testing was performed according to the Clinical Laboratory Standards Institute (CLSI) guidelines using the Kirby–Bauer agar diffusion or microdilution method (CLSI, 2022). Standard strains of *Escherichia coli* ATCC 25922 and *Pseudomonas aeruginosa* ATCC 27853 were used for quality control. Susceptibility testing was conducted against ampicillin, ceftiofur, cefquinome, gentamicin, neomycin, kanamycin, spectinomycin, sulfisoxazole, trimethoprim–sulfamethoxazole, doxycycline, enrofloxacin, chloramphenicol, florfenicol, tilmicosin, tiamulin, and colistin (according to the CLSI guidelines, the microdilution method is acceptable, and disk diffusion methods should not be performed for colistin). These antibiotics are commonly used as veterinary drugs (CSAMR, 2019). CLSI (2018, 2022) and European Committee on Antimicrobial Susceptibility Testing EUCAST (2022) criteria only provide minimum inhibitory concentration (MIC) for gentamicin, sulfisoxazole, doxycycline, and chloramphenicol. For the remaining 12 antibiotics, zone diameter and MIC breakpoints were not provided, and thus, the results are presented as zone diameter or MIC (colistin) values.

### Assessment of the effect of isolated strains on boar semen

The isolated strains were cultured in tryptic soy broth (TSB) (BD, Spark MD, USA) at 27°C or 37°C for 16 h in a shaking bath to assess the pathogenicity of the isolated bacteria on boar sperm. Then, strains were inoculated into the extended semen. The commercial extended semen doses (Yangxiang, Guigang, China), with a concentration of 2.5 × 10^7^ spermatozoa/ml, were collected from the healthy boar and divided into seven aliquots of 8 ml each. One aliquot was used as a control (non-infected samples), while the others were infected with isolated strain at the following initial bacterial concentrations (day 0): 2 × 10^2^, 2 × 10^4^, and 2 × 10^6^ CFU/ml. Non-infected and infected samples were stored in sealed tubes at 17°C for 12 days. TSM was measured using the CASA system (HTR-IVOS II, Hamilton Thorne Research, Beverly, MA, United States) at 0, 2, 4, 6, 8,10, and 12 days. Simultaneously, bacterial growth was evaluated at each infectious dose and time point using plate culture with tryptic soy agar (TSA) (BD, Spark MD, United States).

### Whole-genome sequencing, annotation, and gene prediction

The genome was sequenced using a combination of Illumina NovaSeq6000 (Illumina, San Diego, CA, USA) and Nanopore PromethION sequencing platforms (Oxford Nanopore Technologies, Oxford, UK). The original image data were converted into sequence data by base calling, resulting in raw reads that were saved as a FASTQ file with read sequences and quality information. After removing low-quality data using quality information statistics, the reads were assembled into a contig to generate a complete genome with seamless chromosomes and plasmids using CAUN (version 1.6) and the Hierarchical Genome Assembly Process (HGAP) (Chin et al., 2013; Koren et al., 2017). Finally, the PacBio assembly results were corrected using Illumine reads.

Glimmer (version 3.02, http://ccb.jhu.edu/software/glimmer/index.shtml) and GeneMarkS (version 4.3, http://topaz.gatech.edu/GeneMark) were used for CDS prediction (Besemer et al., 2001; Delcher et al., 2007). tRNA-scan-SE (version 2.0, http://trna.ucsc.edu/software/) was used for tRNA prediction (Chan and Lowe, 2019). Barrnap (https://github.com/tseemann/barrnap) was used for rRNA prediction.

The predicted CDSs were annotated from NCBI non-redundant (NR) (ftp://ftp.ncbi.nlm.nih.gov/blast/db/), Swiss-Prot (https://web.expasy.org/docs/swiss-prot_guideline.html), Protein families (Pfam) (http://pfam.xfam.org/), Gene Ontology (GO) (http://geneontology.org/), Clusters of Orthologous Groups (COG) (https://www.ncbi.nlm.nih.gov/research/cog/), and Kyoto Encyclopedia of Genes and Genomes (KEGG) (https://www.genome.jp/kegg/) databases using Blast2go (version 2.5, https://www.blast2go.com/), Diamond (version 0.8.35, https://github.com/bbuchfink/diamond), and HMMER (version 3.1, http://www.hmmer.org/) sequence alignment tools. Each set of query proteins was aligned with the databases, and the annotations of best-matched subjects (e-value <10^−5^) were obtained for gene annotation.

### Genome-based species identification

To carry out subsequent comparative genomic analyses, we performed species identification based on whole-genome sequence using the online Type (Strain) Genome Server (TYGS) (https://tygs.dsmz.de/) (Meier-Kolthoff and Goeker, 2019) and JSpeciesWS (version3.9.8, https://jspecies.ribohost.com/jspeciesws/#analyse) (Richter et al., 2016).

### Antibiotic resistance genes and comparative genomics analysis

Antibiotic resistance genes were annotated and identified using the online Resistance Gene Identifier (RGI) (version 6.0.1, https://card.mcmaster.ca/analyze/rgi) (select criteria: “Perfect, Strict and Loose hits,” Exclude nudge, High quality/coverage) (Alcock et al., 2022). The ResFinder software (version 4.1, https://cge.food.dtu.dk/services/ResFinder-4.1/) program (identity ≥ 80%, coverage ≥ 60%) within ResFinder data was used to identify the acquired antimicrobial resistance genes. Based on the average nucleotide identity (ANI) data and the phylogenetic tree, the closest homologs to the isolated strain were selected for resistance gene prediction and comparative analysis.

### Data analysis

The data were analyzed using GraphPad Prism 8.0 (San Diego, CA, USA). The data from three independent experiments were presented as means and standard deviations (SD). TSM was compared by Duncan's multiple range tests using one-way analysis of variance (ANOVA) when the F-value was significant (*p* < 0.05). The correlation among the above-mentioned methods was evaluated by linear regression analysis.

### Data availability

Whole-genome sequencing data of the *P. GXZC* strain were submitted to the NCBI (Pseudomonas sp. GXZC, assembly accession: GCF_026967615.1). The BioProject number for the complete genome sequence was PRJNA909457.

## Result

### *Pseudomonas* spp. was the dominant genus in the later stages of semen storage

**Figure 2:**
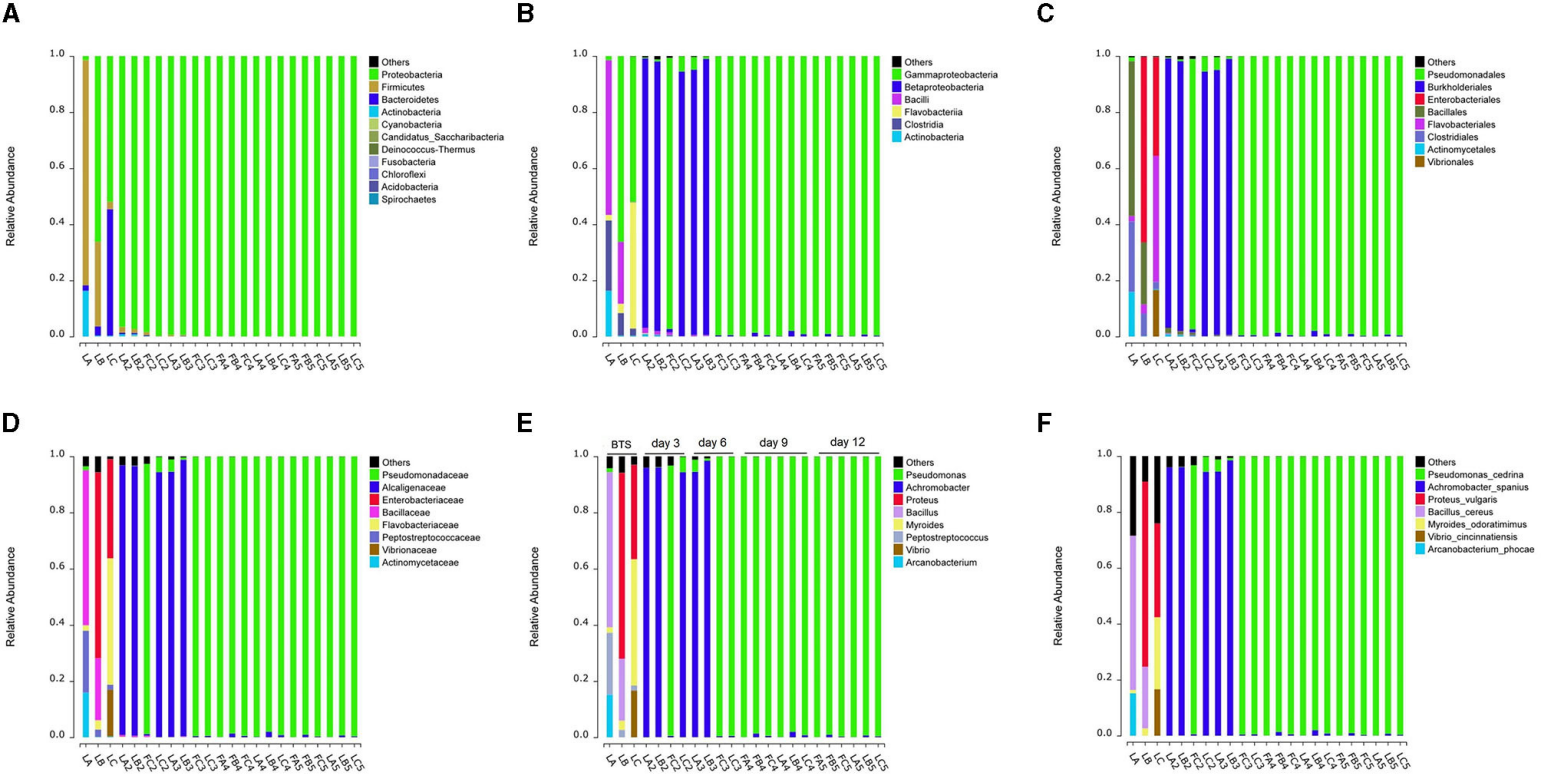
Variations in bacterial community composition of extended semen stored for days. **(A–E)** Shifts in bacterial taxonomic compositions at several taxonomic levels, including phylum **(A)**, class **(B)**, order **(C)**, family **(D)**, genus **(E)**, and species **(F)**. “LA, LB, and LC” are samples that the ejaculate was diluted in sterile BTS. All remaining are samples that the ejaculate was diluted in Zenolong^®^. “FC2, FC3, FA4, FB4, FC4, FA5, FB5, and FC5” are Group I in which the samples were stored at 17°C for the right time (3, 6, 9, or 12 days). Moreover, “LA, LB, LC, LA2, LB2, LC2, LA3, LB3, LC3, LA4, LB4, LC4, LA5, LB5, and LC5” are Group II in which the samples were stored in an incubator under shaking condition with 180 rpm at 37°C for 24 h after having 17°C storages. A, B, and C represent three boars, respectively; numbers 2, 3, 4, and 5 represent 3, 6, 9, and 12 days, where the extended semen was stored. “F” and “L” represent groups I and II, respectively.

After 6 months of investigation in a group with approximately 10,000 boars from three clusters of AI stations distributed throughout China, we observed a high percentage of *Pseudomonas* isolates and flora composition during the late storage stage (storage time > 9 days) of extended boar semen (data not shown). The three randomly selected boars were consistent with the previous background investigation. The extended semen of three boars also showed similar results, with a rapid decrease in total sperm motility (TSM) (Supplementary Figure S1) and an increased proportion of *Pseudomonas* spp. with increasing storage time (Figure 2).

Due to the initially low bacterial levels present in semen during the early stage of semen storage, 12 samples contained <0.3 ng/μl of genomic DNA and were discarded (Supplementary Table S2). To better comprehend the diversity of bacterial composition, the collected semen was incubated at a temperature of 37°C to facilitate rapid bacterial growth. By utilizing 16S rRNA high-throughput sequencing, we were able to partially elucidate the bacterial population, particularly in the extenders without antibiotics (BTS extender), which can be notably more complex. In conclusion, the community diversity decreased after the addition of antibiotics and after 12-day storage at 17°C (Supplementary Figure S2). In the later stages of storage (9 and 12 days), all samples were dominated by *Pseudomonas* spp. (relative abundance: 99.05%−99.86%), followed by *Achromobacter* spp. (relative abundance: 0.06%−1.95%) (Figure 2E, Supplementary Table S3).

### *P. GXZC* strain identified as a strain of *Pseudomonas fluorescens* group with adaptation to 17°C temperature and multidrug resistance

All colony morphologies of samples inoculated on TSA were visually consistent at the same temperature. However, colony morphologies were different between 17°C and 37°C. Sequence alignment exhibited that the isolates stored at 17°C were all *Pseudomonas* spp. The sequences shared 100% identity, designated as *P. GXZC* strain. Similarly, the isolates stored at 37°C were all *Achromobacter* spp., and the sequences shared 100% identity, designated as *A. GXZC* strain. No other bacteria were isolated. However, it could not identify the groups or species using only 16S rRNA sequence comparisons. Multilocus sequence typing (MLST) was performed to identify the groups and species of *Pseudomonas* spp. using combined 16S rRNA, *gyrB, rpoD*, and *rpoB* sequences (Ait Tayeb et al., 2005; Mulet et al., 2009; Edgar, 2010), and *Achromobacter* spp. were identified based on 16S rRNA and *recA* sequences (Gomila et al., 2014). The sequencing primers are shown in Supplementary Table S4. *P. GXYX* was identified as *P. azotoformans* (with a threshold of 97% similarity for the species) within *P. fluorescens* group (Mulet et al., 2010), and *A. GXYX* was identified as *A. xylosoxidans* (with a threshold of 98% similarity for the species) (Gomila et al., 2014).

**Figure 3:**
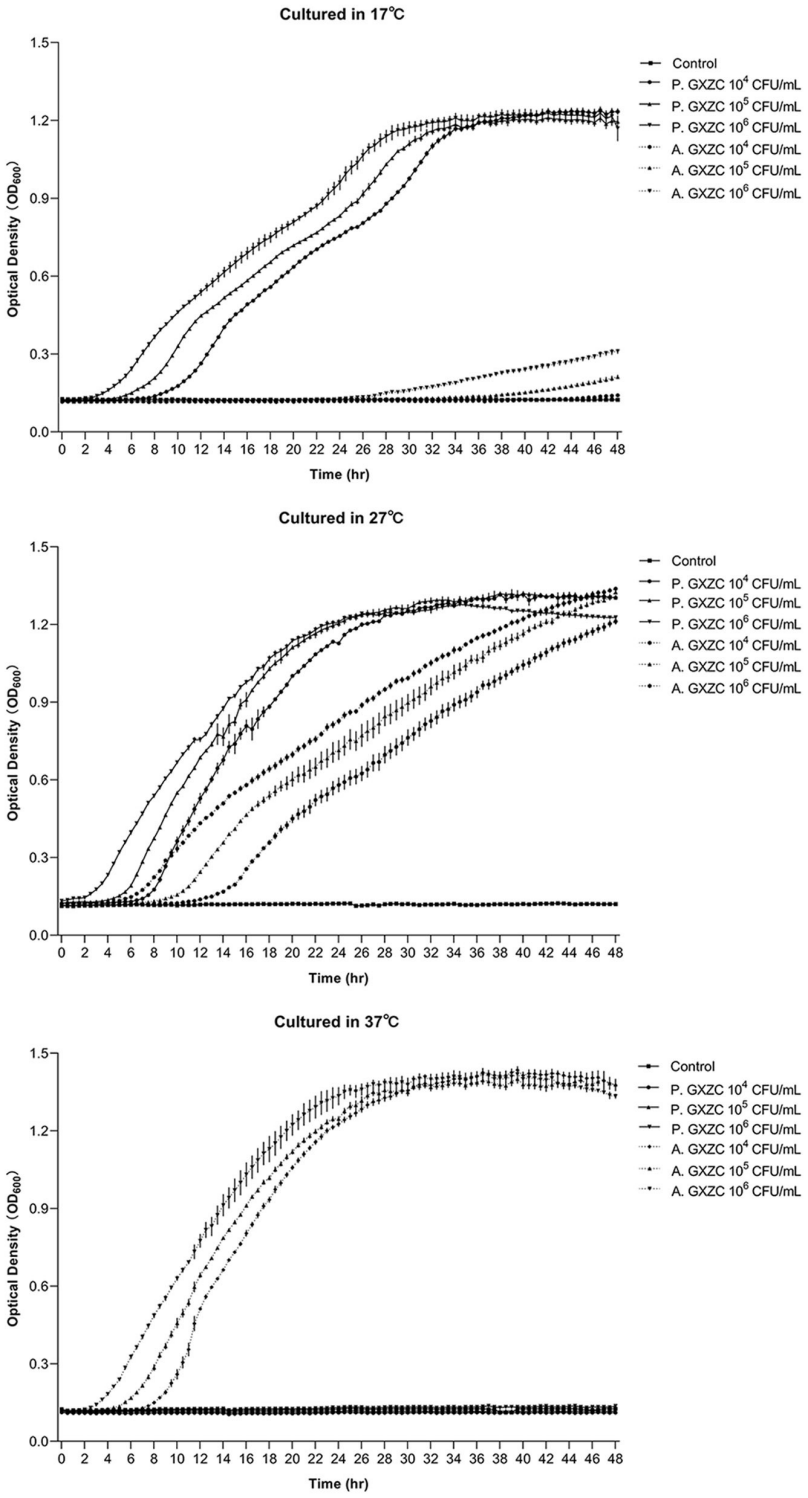
Bacterial proliferation curve at different temperature. The proliferation curve of *P. GXZC* and *A. GXZC* with inoculation of three 10-fold ratio gradient concentrations at 17°C, 27°C, and 37°C, respectively. Data are shown as mean ± SD. n = 3.

*P. GXZC* and *A. GXZC* had significantly different proliferation curves at 17°C, 27°C, and 37°C, respectively (Figure 3). At 17°C, *P. GXZC* proliferated more actively than *A. GXZC*. The strain entered the log phase from 2 to 8 h and then the stationary phase from 28 to 34 h, inoculating three 10-fold ratio gradient concentrations. Conversely, *A. GXZC* proliferation entered the log phase at the later stages of the experiment and did not enter the stationary phase until the end time. However, at 37°C, *P. GXZC* could not proliferate, while *A. GXZC* proliferation entered the log phase from 2 to 8 h and the stationary phase from 24 to 30 h. Although both isolated strains proliferated actively at 27°C, *P. GXZC* exhibited faster proliferation than *A. GXZC*, with an earlier point of entering the log and stationary phases.

Antimicrobial susceptibility testing showed that *P. GXZC* strain exhibited resistance to gentamicin, sulfisoxazole, doxycycline, and chloramphenicol (Table 1). For the 12 antibiotics lacking zone diameter or MIC breakpoints, only zone diameter or MIC values are provided (referring to the criteria of *P. aeruginosa, P. GXZC* strain could identify resistance to colistin).

**Table 1:** Antimicrobial susceptibility testing.

**Antimicrobial agent**	***P. GXYX***	***A. GXYX***
*Microdilution method*	*MIC (μg/mL)*	
Gentamicin	R	R
Sulfisoxazole	R	R
Doxycycline	R	S
Chloramphenicol	R	S
Colistin	2048	32
*Kirby–Bauer agar diffusion method*	*Inhibition zone diameter (mm)*	
Ampicillin (10 μg)	0	26
Ceftiofur (30 μg)	0	0
Cefquinome (30 μg)	0	0
Gentamicin (10 μg)	0	0
Neomycin (30 μg)	10	14
Kanamycin (30 μg)	0	0
Spectinomycin (100 μg)	4	5
Sulfisoxazole (300 μg)	0	0
Trimethoprim–Sulfamethoxazole (1.25/23.75 μg)	0	0
Doxycycline (30 μg)	0	17
Enrofloxacin (10 μg)	0	14
Chloramphenicol (30 μg)	0	25
Florfenicol (30 μg)	0	27
Tilmicosin (15 μg)	0	0
Tiamulin (30 μg)	0	0

^a^R, Resistant was designated using suggested MIC breakpoints (gentamicin, ≥ 16 μg/ml; sulfisoxazole, ≥ 512 μg/ml; doxycycline, ≥ 16 μg/ml; chloramphenicol, ≥32 μg/ml) from CLSI reference for non-Enterobacteriaceae.

^b^S, Susceptible was designated using suggested MIC breakpoints (doxycycline, ≤ 4 μg/ml; chloramphenicol, ≤ 8 μg/ml) from CLSI reference for non-Enterobacteriaceae.

### The *P. GXZC* strain harmed sperm vitality in extended semen storage

**Figure 4:**
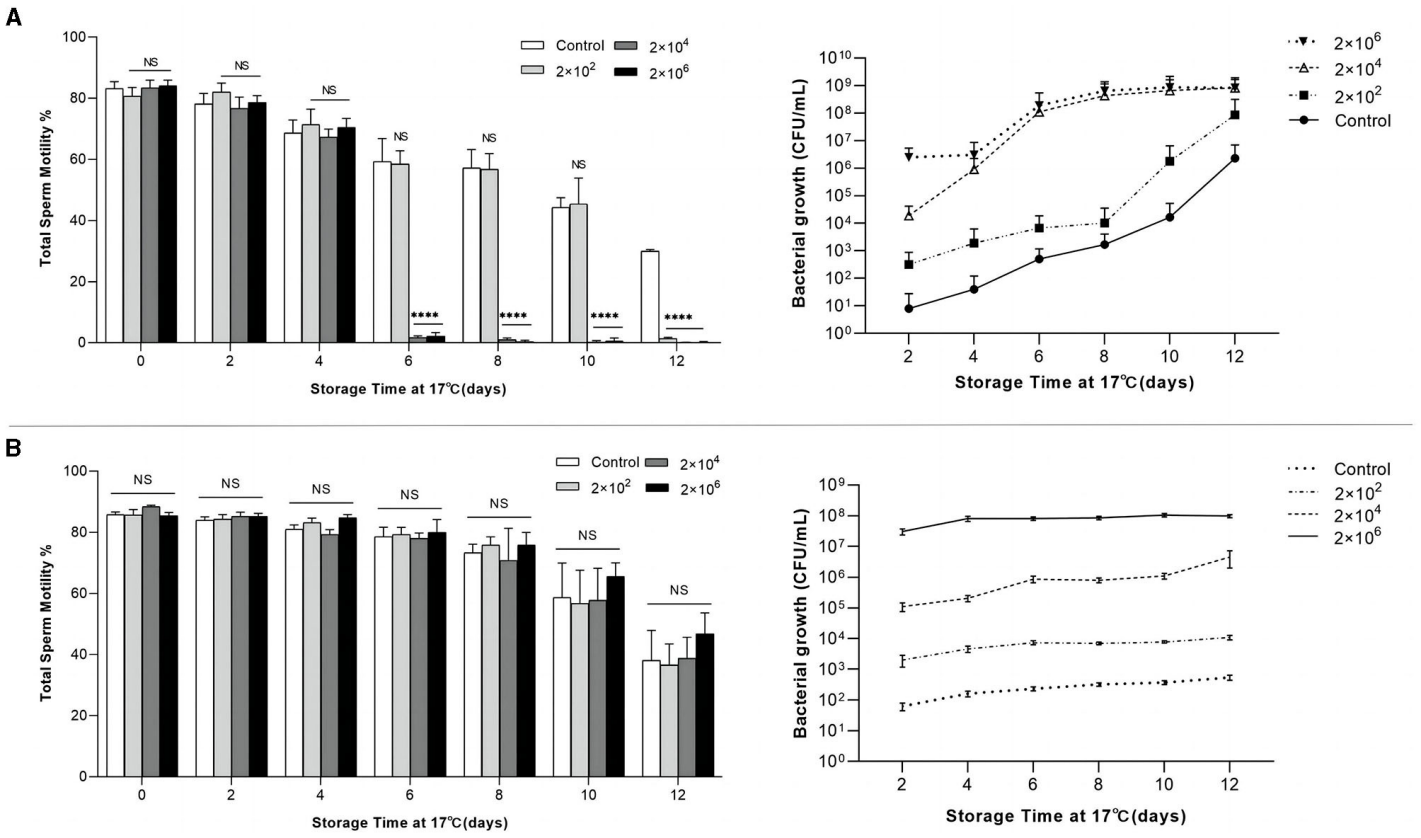
Relation of total sperm motility and bacterial growth in extended semen. Total sperm motility of extended semen incubated with different infectious concentrations (2 × 10^2^, 2 × 10^4^, and 2 × 10^6^ CFU/ml) over 12 days of storage at 17°C, and bacterial growth plotted by colonies counted directly after incubating the TSA plates at 28°C for 24 h. **(A)** Incubated with P. GXYX strain. **(B)** Incubated with A. GXYX strain. Data are shown as mean ± SD. *n* = 3. “NS”: no significant; ^****^p < 0.0001.

A study has confirmed a positive correlation between pregnancy rate and semen motility (Lucca et al., 2021). Herein, the effect of *P. GXZC* strain on the vitality of sperm was determined. The growth dynamics of the *P. GXZC* and *A. GXZC* strains are shown in Figure 4 and are notably different during the 12-day storage period at 17°C. Consistent with the assessment results above, *P. GXZC* proliferates actively in extended semen at 17°C (Figure 4A). All groups inoculated with different concentration of *P. GXZC* strain reach the stationary phase at approximately 10^8^ CFU/ml in liquid extended semen. The proliferation of the *P. GXZC* strain adversely affected sperm vitality in the later stages of extended semen storage. On day 6, the tube containing 2 × 10^6^ CFU/ml or 2 × 10^4^ CFU/ml of *P. GXZC* differed significantly from the negative control. On day 12, all treatments differed from the control. It seems that only when the bacteria proliferate to a certain concentration (> 10^6^ CFU/ml), they show significant negative effects on sperm vitality. In contrast, *A. GXZC* proliferation was extremely slow in liquid extended semen at 17°C. Moreover, there were no differences (Figure 4B) in the TSM between the treatments and the negative control over the entire time.

### Genomic characteristics of *P. GXZC* strain

**Figure 5:**
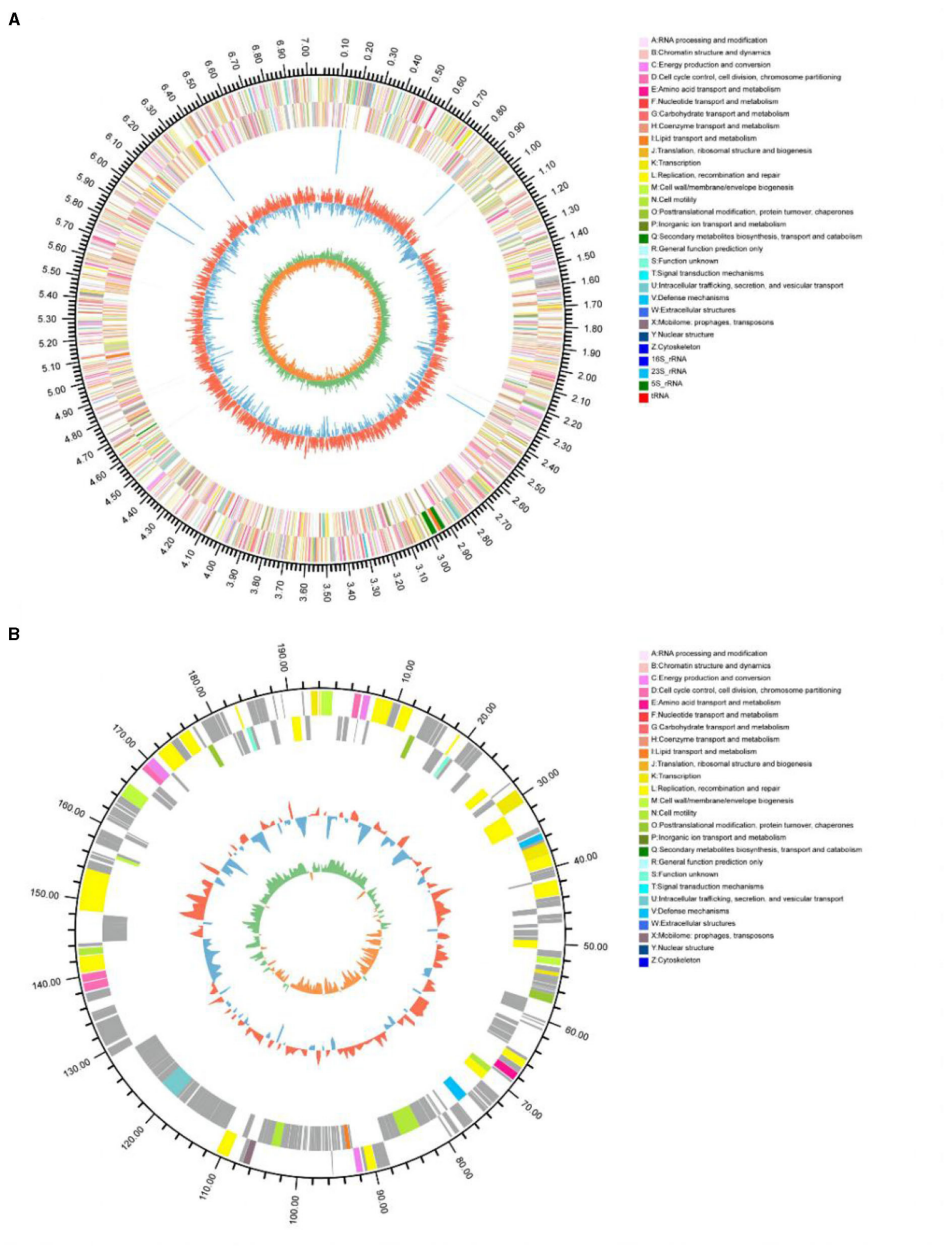
Circular representation of the *P. GXZC* genome structure. **(A)** Circular representation of chromosome; **(B)** Circular representation of plasmid. The outermost circle of the diagram is the genome size identification; the second and third circles are CDS on positive and negative strands, and different colors indicate the functional classification of different COGs of CDS; the fourth circle is rRNA, tRNA; the fifth circle is GC content, the outer red part indicates that the GC content of the region is higher than the (average GC content of the whole genome, and the higher the peak indicates the larger the difference with the average GC content The innermost circle is the GC-Skew value, the specific algorithm is G-C/G+C.

The general characteristics of the P. GXZC strain genome are shown in Table 2, which consisted of 7,253,751 base pairs with an average G + C content of 60.23%. The genome contained approximately 6,790 predicted CDSs and 73 tRNA and 20 rRNA genes (8, 5S; 6, 16S; and 6, 23S) (Figure 5). The CDS numbers allocated to the different databases are presented in Table 2.

**Table 2:** Genomic properties of P. GXZC strain.

**Characteristics**	**Value**
Genome size (bp)	7,253,751
GC content (%)	60.23
Topology	Circular
Chromosome size (bp)	7,059,625
Plasmid size (bp)	194,126
Chromosome GC content (%)	60.39
Plasmid GC content (%)	54.28
Chromosome	1
Plasmid	1
tRNA	73
rRNA (5S,16S,23S)	20
CDS (chromosome, plasmid)	6,790
Genes assigned to NR	6,765
Genes assigned to Swiss-Prot	4,902
Genes assigned to COG	5,409
Genes assigned to KEGG	2,217
Genes assigned to GO	4,756
Genes assigned to Pfam	5,691

### Species determination of *P*. *GXZC* based on whole-genome sequencing

**Figure 6:**
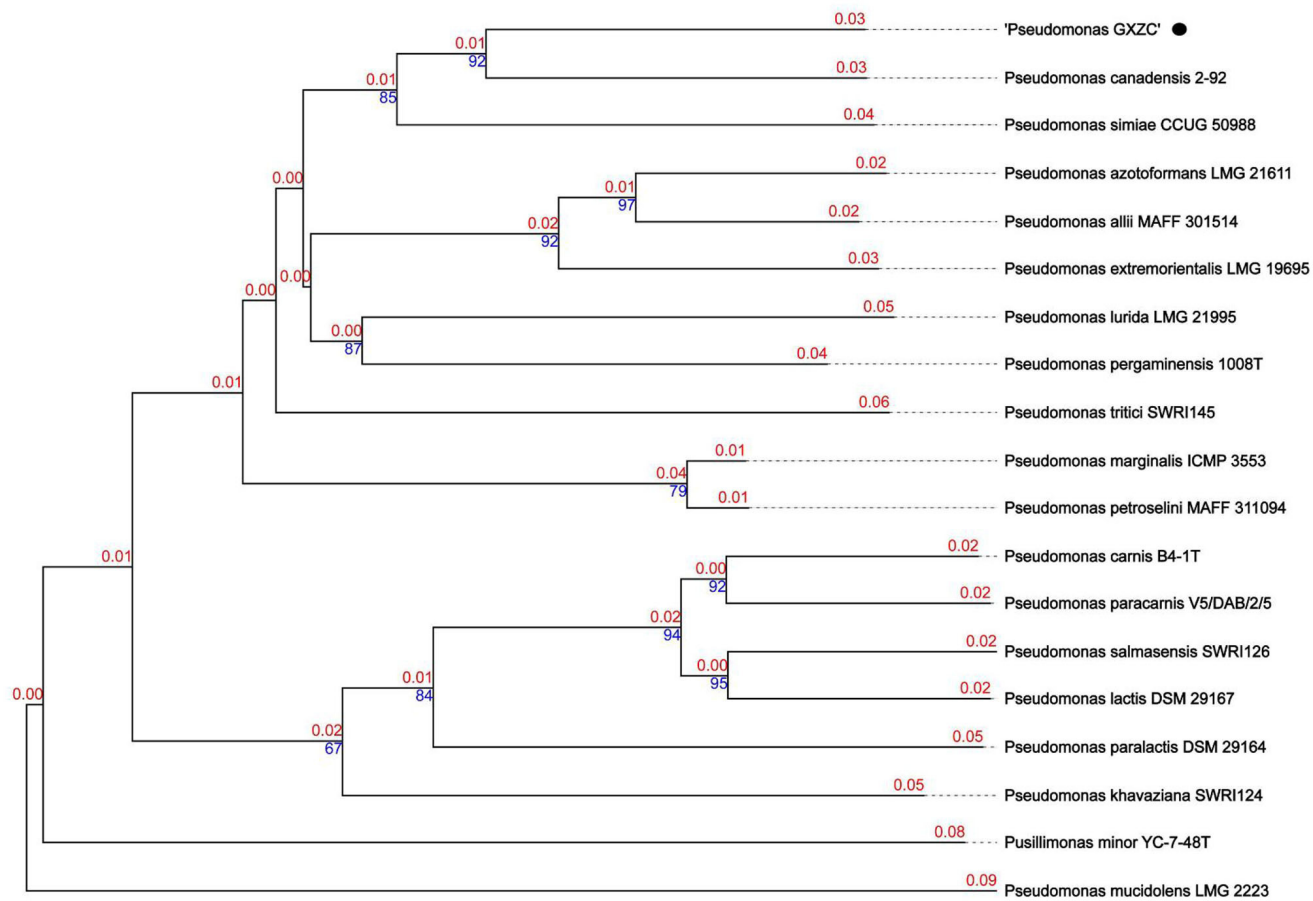
Phylogenetic relationships of *P. GXZC* whole genome was conducted in Type (Strain) Genome Server (TYGS) (date: 02 March 2023).

*P. GXZC* strain does not belong to any species in the Type (Strain) Genome Server (TYGS) database and is potentially a new species in *P. fluorescens* group. The closest type strain genomes are *Pseudomonas canadensis* Feb-92 strain (assembly accession: GCF_026967615.1) and *Pseudomonas simiae* CCUG 50988 (assembly accession: GCF_900111895.1) (Figure 6).

The best-matching type strain is *P. canadensis* Feb-92 (assembly accession: GCF_026967615.1, type strain), with an average nucleotide identity based on BLAST+ (ANIb) data of 92.4% using the JSpeciesWS (Supplementary Table S5). We selected the four best-matching type strains with ANIb > 90%, namely, *P. canadensis* Feb-92 (assembly accession: GCF_000503215.1), *P. canadensis* PA-6-2A (assembly accession: GCF_021605905.1), *P. simiae* CCUG 50988 (assembly accession: GCF_900111895.1), and *P. simiae* PCL1751 (assembly accession: GCF_000934565.1), to conduct a comparative analysis with *P. GXZC*.

### Comparative analysis of antibiotic resistance genes (ARGs)

Based on the phylogenetic tree and ANIb, the four *Pseudomonas* strains conducted a comparative analysis with *P. GXZC*. ARGs of all strains were predicted and categorized by drug classes using online RGI 6.0.1. All strains were predicted diverse and large drug-resistant genes (Table 3). However, the *GXZC* strain has more ARG hits than other comparison strains with the following drug classes: aminoglycoside antibiotic (with perfect or strict criteria only), tetracycline antibiotic, fluoroquinolone antibiotic, macrolide antibiotic, penam, cephalosporin, disinfecting agents and antiseptics, sulfonamide antibiotic, nitroimidazole antibiotic, and lincosamide antibiotic. Except for penam, all drug classes are widely used in pig farms.

**Table 3:** Summary of predicted ARGs categorized by drug classes using Resistance Gene Identifier (RGI).

**RGI criteria^a^**	**Drug class**	***P*.*GXZC*^b^**	***P. canadensis* PA-6-2A**	***P. canadensis* Feb-92**	***P. simiae* CCUG 50988**	***P. simiae* PCL1751**
Perfect	**Aminoglycoside antibiotic**	3	0	0	0	0
Strict	**Aminoglycoside antibiotic**	1	0	0	0	0
	Cephalosporin	1	1	1	1	1
	Disinfecting agents and antiseptics	1	1	1	1	1
	Fluoroquinolone antibiotic	5	6	5	5	5
	Glycopeptide antibiotic	1	1	1	1	1
	Glycylcycline	1	1	1	1	1
	Penam	1	1	1	1	1
	Phenicol antibiotic	1	1	1	1	1
	Phosphonic acid antibiotic	1	1	1	0	0
	Rifamycin antibiotic	1	1	1	1	1
	Tetracycline antibiotic	5	5	4	1	4
Loose	**Tetracycline antibiotic**	187	181	178	171	169
	**Fluoroquinolone antibiotic**	185	180	170	163	160
	**Macrolide antibiotic**	142	139	137	133	129
	**Penam**	113	111	108	106	102
	Aminoglycoside antibiotic	94	98	98	81	83
	Peptide antibiotic	78	74	73	69	69
	Phenicol antibiotic	77	77	68	69	68
	**Cephalosporin**	68	67	64	61	61
	**Disinfecting agents and antiseptics**	67	62	57	58	58
	Cephamycin	55	56	54	50	50
	Carbapenem	48	50	49	43	43
	Aminocoumarin antibiotic	38	43	46	41	40
	Diaminopyrimidine antibiotic	35	37	32	33	33
	Monobactam	34	36	35	33	33
	Penem	28	30	29	28	28
	Glycopeptide antibiotic	27	28	28	27	27
	**Sulfonamide antibiotic**	13	10	12	12	12
	Rifamycin antibiotic	12	15	13	14	14
	Glycylcycline	10	10	8	10	10
	**Nitroimidazole antibiotic**	8	7	7	7	7
	**Lincosamide antibiotic**	7	6	6	6	6
	Nucleoside antibiotic	7	6	6	5	5
	Bicyclomycin-like antibiotic	5	6	6	5	6
	Antibacterial free fatty acids	4	3	3	4	4
	Oxazolidinone antibiotic	4	4	4	4	4
	Pleuromutilin antibiotic	4	4	4	4	3
	Elfamycin antibiotic	3	4	4	4	4
	Fusidane antibiotic	3	3	3	3	3
	Mupirocin-like antibiotic	3	3	3	3	3
	Phosphonic acid antibiotic	3	15	16	13	13
	Streptogramin antibiotic	3	3	4	3	4
	Isoniazid-like antibiotic	2	4	5	2	2
	Polyamine antibiotic	1	1	1	1	1
	Salicylic acid antibiotic	1	1	1	1	1
	Streptogramin A antibiotic	1	1	2	2	2
	Streptogramin B antibiotic	1	1	1	1	1

^a^RGI Criteria: “Perfect, Strict, Loose” have been mentioned in the published articles. A more technical definition of these terms refers to this website (https://github.com/arpcard/rgi/issues/140).

^b^Plasmid sequences of the P. GXZC strain were not predicted ARG hits using the same criteria in CARD.

### Antimicrobial resistance island of *P. GXZC*

The study identified nine acquired AMR genes conferring resistance to three drug classes of antibiotics in the chromosome of the *GXZC* strain using ResFinder 4.1 (Table 4). Four other close-type strains were unidentified. *P. GXZC* strain accepted nine acquired AMR genes conferring resistance to aminoglycoside antibiotics. Aminoglycosides are the most commonly used antibacterial additives in extended boar semen.

**Table 4:** Acquired antibiotic resistance genes predicted using ResFinder 4.1.

**Class**	**Resistance gene**	**Phenotype**	**Location**	**Gene position**	**Identity (%)**	**GI position**
Aminoglycoside	aph(3”)-Ib	Streptomycin	Chromosome	398950–399753		(364961–446597)
	aph(6)-Id	Streptomycin		405693–406436	100.00	
	aph(3”)-Ib	Streptomycin		406529–407332	100.00	
	aph(3')-Ia	Neomycin, Kanamycin, Lividomycin, Paromomycin, Ribostamycin		404542–405357	99.88	
	aph(6)-Id	Streptomycin		398114–398857	99.87	
Folate pathway antagonist	sul1	Sulfamethoxazole		415230–415844	100.00	
	sul1	Sulfamethoxazole		423055–423581	100.00	
Tetracycline	tet(G)	Doxycycline, Tetracycline		419498–420673	100.00	
Aminoglycoside	aac(3)-IId	Apramycin, Gentamicin, Tobramycin, Dibekacin, Netilmicin, Sisomicin	Chromosome	7023795–7024655	99.88	(70181560–7044848)

**Figure 7:**
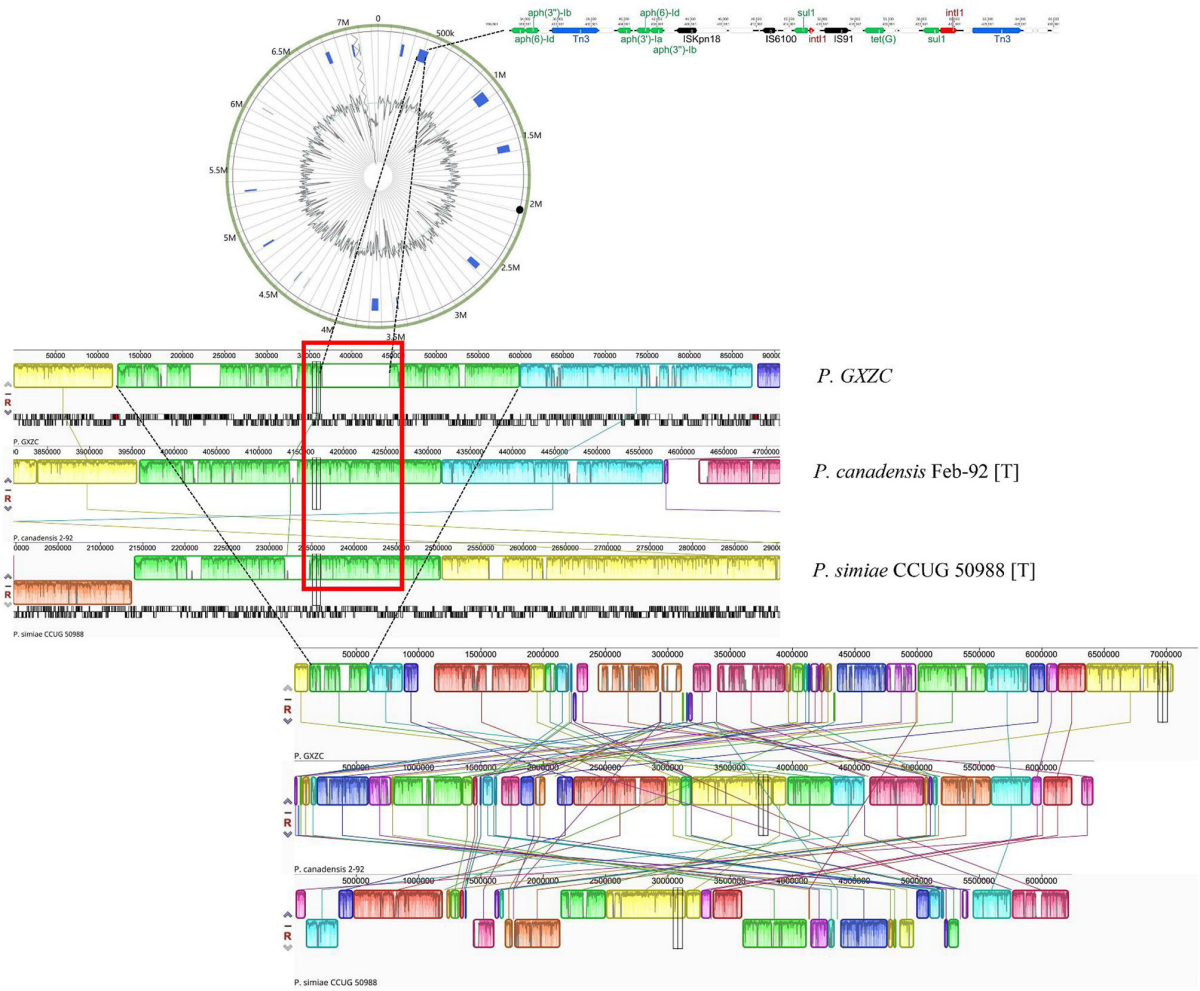
Antimicrobial resistance island of *P. GXZC* strain. Whole-genome alignment of *P. GXZC*, with two types of strains, *P. canadensis* Feb-92 and *P. simiae* CCUG 50988, is conducted using MAUVE. At the homologous site (in the red box), *P. GXZC* strain has an approximately 81kb insertion which is identified as an antimicrobial resistance island.

Using IslandViewer 4 with the IslandPath-DIMOB method (Bertelli et al., 2017), we identified 15 gene islands (GI) in the chromosome. One acquired AMR is located in one GI. The other eight acquired AMR genes were clustered in another 81.6-kb GI. This GI contains three types of mobile genetic elements, two integrons, three IS elements, and two transposons (Figure 7). This antimicrobial resistance island structure represents the potential for rapidly acquiring resistance markers under antimicrobial pressure. Alignment with MAUVE (version 1.1.3) revealed that similar GI structures are absent at the homologous location of the other two best-matching type strains (Figure 7).

## Discussion

Our study reviewed previous experiments on the assessment of semen quality during long-term preservation (Huo et al., 2002; Dube et al., 2004; Bussalleu et al., 2017; Shaoyong et al., 2019; Li et al., 2022), and we found that sperm motility analysis is the most widely used and effective method. In commercial extended semen production, TSM analysis performed by CASA is essential for quality control (Amann and Waberski, 2014). To simplify the evaluation of our experiments and facilitate statistical analysis, TSM analysis was exclusively used in our research.

Although various factors contribute to semen deterioration in a commercial extender containing antibiotics during extended storage, microbial contamination is a significant risk factor. High-throughput sequencing revealed dynamic bacterial proliferation during extended semen storage. *Pseudomonas* spp. was observed to gradually become the dominant flora in 12-day long-term semen storage. The result appears to be relative to an environment of 17°C and antibiotic supplements.

*Pseudomonas* is one of the most complex bacterial genus and comprises the largest number of gram-negative bacteria species (Gomila et al., 2015). Many isolations initially identified as “*P. fluorescens* species” are now reclassified as “*P. fluorescens* species complex” (Scales et al., 2014) or *P. fluorescens* intrageneric groups based on MLST (Mulet et al., 2010). MLST is a rapid genotyping method, but it has low resolution and is insufficient for species typing for *P. fluorescens* group to perform comparative genomic analysis. With the phylogenetic tree and ANIb database on WGS (Richter et al., 2016), *P. GXZC* strain was identified as a potential new species in *Pseudomonas fluorescens* group eventually. WGS is a gold standard method to identify *Pseudomonas* species (Tohya et al., 2022).

Most members of the *P. fluorescens* species complex (from the environment) optimum growth temperature are <30 °C, and their growth decreases as temperature rises above 30°C (Buchon et al., 2000; Zhang et al., 2019). This species complex is the most frequently reported “psychrotrophic bacteria” in cold-stored raw fluid milk and deteriorates stored milk (Craven and Macauley, 1993; Shah, 1994; Wiedmann et al., 2000; Dogan and Boor, 2003; Gunasekera et al., 2003; Du et al., 2022). It was the primary isolated strain among all contaminants in some studies (Dogan and Boor, 2003; de Oliveira et al., 2015; Du et al., 2022). The storage environment of the extended boar semen appears to be similar to that of raw fluid milk. In our study, *P. GXZC* actively proliferated at 17°C and 27°C but did not proliferate at 37°C and entered a viable-but-not-culturable (VBNC) state (Bunker et al., 2004). This may be one of the main reasons for *P. GXZC* to be the dominant flora in the 12-day storage of extended semen experiment.

We first confirmed that the isolated strain of *P. fluorescens* had a negative effect on sperm vitality when the bacteria proliferated to a certain concentration (> 10^6^ CFU/ml). The negative effect of *P. fluorescens* on sperm motility is concentration-dependent. The characteristic may be related to sperm damage caused by bacterial outer membrane vesicles (OMVs) or lipopolysaccharide (LPS) (He et al., 2017; Gao et al., 2018) and the reduction in sperm protein phosphorylation levels (Sepulveda et al., 2016).

Pasteurization (70–80°C) can treat refrigerated raw milk, but for extended semen, antibiotics are one of the few options to inhibit bacterial proliferation in extended semen. However, antimicrobial susceptibility testing demonstrated that *P. GXZC* developed a high level of resistance to almost all commonly used veterinary antibiotics, including colistin, a the last resort for combating multidrug-resistant Gram-negative bacteria (MDR-GNB) (Singhal et al., 2022).

As a member of the species complex, *P. GXZC* strain has 6,790 predicted CDSs and diverse mechanisms of drug resistance. Numerous resistant phenotypes in this complex were related to intrinsic, adaptive, and acquired antimicrobial resistance mechanisms (Silverio et al., 2022). Whole-genome sequencing has revealed that the *P. GXZC* strain has more drug-resistant genes than closely related strains (identified based on WGS), which may be related to antibiotic use in extended boar semen or treating boars. Although previous studies have reported that transferable resistance mechanisms are rare in this complex, we confirmed horizontal gene transfer (HGT) of antimicrobial resistance in *P. GXZC* (Silverio et al., 2022). The mechanism of antibiotic resistance includes reduced permeability to antibiotic, antibiotic efflux, antibiotic inactivation, and antibiotic target alteration (Chopra and Roberts, 2001; Zeng and Jin, 2003; Xiao and Hu, 2012; Ashenafi et al., 2014; Chung et al., 2015; Kapoor et al., 2017). The resistance island with eight clustered resistance genes and various mobile genetic elements may indicate that these strains acquire resistance genes more rapidly than previously assumed. Our results suggest that adding antibiotics seems difficult to resolve the multidrug-resistant *P. fluorescens* strains and is an unsustainable approach to microbial control in extended semen.

*P. fluorescens* species complex are ubiquitous microorganisms in the environment (Scales et al., 2014). Although several previous studies have identified the presence of *P. fluorescens* in boar semen, the risks it poses in boar semen storage have not been taken seriously. This may be attributed to the past practice of storing extended semen for a short period, during which bacteria did not have sufficient time to proliferate and cause significant damage to sperm. However, with the growing adoption of commercial long-term extended semen which semen storage time shift from shorter storage durations to longer periods, the adverse effects of bacterial proliferation on extended semen storage should be given more attention. The biological characteristics exhibited by *P. GXZC*, as explored in this study, may pose a challenge for long-term semen storage. Several studies have attempted boar semen storage at 5°C in the absence of antibiotics (Paschoal et al., 2020; Jakel et al., 2021), which seem to similarly consider the impact of these cold-adapted proliferating or psychrotrophic bacteria.

### Conclusion

Our study identified a potential new species *GXZC* strain in *P. fluorescens* group as the dominant flora in extended boar semen. This strain exhibited adverse effects on sperm quality. The strain can better adapt to growth at a moderately low temperature (17°C) and has multidrug resistance. Whole-genome sequencing and comparative genomic analysis revealed that the strain developed devise intrinsic resistance and horizontal resistance gene transfer. The unregulated proliferation of multidrug-resistant *P. fluorescens* within commercial extenders presents potential risks to the long-term preservation of extended boar semen at 17°C and can impact the success of artificial insemination (AI) procedures. In AI facilities, heightened attention should be given to the prevalence of this type of bacterial contamination, and more effective antimicrobial and hygienic management strategies should be developed to control it.

## Data availability statement

The datasets presented in this study can be found in online repositories. The names of the repository/repositories and accession number(s) can be found in the article/Supplementary material.

## Ethics statement

The animal study was approved by Laboratory Animal Monitoring Committee of Huazhong Agricultural University. The study was conducted in accordance with the local legislation and institutional requirements.

## Author contributions

ZX: Conceptualization, Data curation, Investigation, Methodology, Writing—original draft, Writing—review and editing. ZH: Writing—review and editing, Data curation. XinL: Writing—review and editing. DG: Writing—review and editing. LW: Writing—review and editing. SL: Data curation, Investigation, Writing—review and editing. JZ: Data curation, Investigation, Writing—review and editing. XiaL: Funding acquisition, Supervision, Writing—review and editing. PQ: Conceptualization, Writing—review and editing.
