# Supplementary material for: The multidrug-resistant Pseudomonas fluorescens strain: a hidden threat in boar semen preservation
Source: Front Microbiol. 2023 Oct 6;14:1279630. doi: 10.3389/fmicb.2023.1279630 (PMC10588451; doi:10.3389/fmicb.2023.1279630)
Supplement: Supplementary file 1 [file Data_Sheet_1.pdf]

Supplemental Information

This file contains

1) Supplementary Tables S1 to S5.

2) Supplementary Figures S1, S2.

3) Supplementary Methods

Table S1. Parameter Settings CASA IVOS (Version 12.0 IVOS Hamilton Thorne Bioscience, Beverly, USA)

Analysis Setup	Setting
Apply sort	0
Frames acquired	60
Frame rate	40 Hz
Minimum contrast	40
Minimum cell size	5 pixels
Minimum static contrast	15
Straightness (STR) Threshold	25%
VAP cutoff	5.0 µm/s
Progressive minimum VAP	10.0 µm/s
VSL cutoff	5 µm/s
Cell intensity	70
Static head size	0.26 to 7.88
Static head intensity	0.14 to 1.99
Static elongation	10 to 98
Slow cells motile	No
Magnification	8.75
Magnification	8.75
Video frequency	60
Bright field	No
LED illumination intensity	2188
IDENT illumination intensity	2598
Temperature, Set	37.5 °C
Chamber depth	10 µm
Chamber position	14.5 µm
Chamber position B	15.5 µm
Chamber position C	16.5 µm
Chamber position D	17.5 µm
Chamber type	Makler
Field selection mode	Auto
IDENT fluorescent mode	OFF
Integrating time	1 Frame

Table S2. The concentration of genomic DNA

Grouping	Boar Code	Semen Storage Time (days)	Sample name	Library construction	Concentration (ng/μL)	Note
Group III	A	0	FA	HiSeq/MiSeq-meta-16S rRNA gene region V3-V4	0.03	Low concentration, Discarded
	A	0	FA1		0.04	Low concentration, Discarded
	A	3	FA2		0.04	Low concentration, Discarded
	A	6	FA3		0.24	Low concentration, Discarded
	A	9	FA4		24.64	
	A	12	FA5		29.8	
	B	0	FB		0.03	Low concentration, Discarded
	B	0	FB1		0.03	Low concentration, Discarded
	B	3	FB2		0.05	Low concentration, Discarded
	B	6	FB3		0.13	Low concentration, Discarded
	B	9	FB4		25.9	
	B	12	FB5		30.86	
	C	0	FC		0.04	
	C	0	FC1		0.03	Low concentration, Discarded
	C	3	FC2		0.86	
	C	6	FC3		31.33	
	C	9	FC4		31.32	
	C	12	FC5		40.66	
Group II	A	0	LA	HiSeq/MiSeq-meta-16S rRNA gene region V3-V4	21.34	
	A	0	LA1		0.2	Low concentration, Discarded
	A	3	LA2		0.39	
	A	6	LA3		4.18	
	A	9	LA4		26.52	
	A	12	LA5		34.48	
	B	0	LB		26.13	
	B	0	LB1		0.12	Low concentration, Discarded
	B	3	LB2		1.56	
	B	6	LB3		5.78	

B	9	LB4	17.56	
B	12	LB5	27.63	
C	0	LC	27.74	
C	0	LC1	0.22	Low concentration, Discarded
C	3	LC2	13.74	
C	6	LC3	25.94	
C	9	LC4	32.4	
C	12	LC5	28.28	

Table S3. Relative abundance of samples at the genus level

**Genus**	LA	LB	LC	LA2	LB2	FC2	LC2	LA3	LB3	FC3	LC3	FA4	FB4	FC4	LA4	LB4	LC4	FA5	FB5	FC5	LA5	LB5	LC5
Arcanobacterium	15.20 %	0.00 %	0.00 %	0.00 %	0.00 %	0.00 %	0.00 %	0.00 %	0.00 %	0.00 %	0.00 %	0.00 %	0.00 %	0.00 %	0.00 %	0.00 %	0.00 %	0.00 %	0.00 %	0.00 %	0.00 %	0.00 %	0.00 %
Vibrio	0.00 %	0.00 %	16.63 %	0.00 %	0.00 %	0.00 %	0.00 %	0.00 %	0.00 %	0.00 %	0.00 %	0.00 %	0.00 %	0.00 %	0.00 %	0.00 %	0.00 %	0.00 %	0.01 %	0.00 %	0.00 %	0.00 %	0.00 %
Peptostreptococcus	22.06 %	2.62 %	1.81 %	0.00 %	0.00 %	0.00 %	0.00 %	0.00 %	0.00 %	0.00 %	0.00 %	0.00 %	0.00 %	0.00 %	0.00 %	0.00 %	0.00 %	0.00 %	0.00 %	0.00 %	0.00 %	0.00 %	0.00 %
Myroides	1.92 %	3.30 %	44.98 %	0.00 %	0.00 %	0.00 %	0.02 %	0.00 %	0.00 %	0.00 %	0.00 %	0.00 %	0.00 %	0.00 %	0.00 %	0.00 %	0.00 %	0.00 %	0.00 %	0.00 %	0.00 %	0.00 %	0.00 %
Bacillus	55.20 %	22.06 %	0.00 %	0.00 %	0.00 %	0.00 %	0.00 %	0.04 %	0.00 %	0.00 %	0.00 %	0.00 %	0.00 %	0.00 %	0.00 %	0.00 %	0.00 %	0.00 %	0.00 %	0.00 %	0.00 %	0.00 %	0.00 %
Proteus	0.01 %	66.18 %	33.56 %	0.00 %	0.05 %	0.00 %	0.00 %	0.00 %	0.00 %	0.00 %	0.00 %	0.00 %	0.00 %	0.00 %	0.00 %	0.00 %	0.00 %	0.00 %	0.00 %	0.00 %	0.00 %	0.00 %	0.00 %
Achromobacter	0.00 %	0.00 %	0.00 %	95.98 %	96.02 %	0.50 %	94.37 %	94.44 %	98.47 %	0.40 %	0.47 %	0.06 %	1.31 %	0.50 %	0.11 %	1.95 %	0.79 %	0.07 %	0.87 %	0.28 %	0.09 %	0.66 %	0.35 %
Pseudomonas	1.40 %	0.03 %	0.05 %	0.03 %	0.12 %	96.23 %	5.42 %	4.33 %	0.84 %	99.54 %	99.43 %	99.85 %	98.58 %	99.46 %	99.80 %	97.97 %	99.13 %	99.86 %	99.05 %	99.66 %	99.82 %	99.26 %	99.62 %
Others	4.21 %	5.81 %	2.97 %	3.99 %	3.80 %	3.27 %	0.19 %	1.19 %	0.69 %	0.07 %	0.10 %	0.08 %	0.11 %	0.04 %	0.09 %	0.07 %	0.08 %	0.07 %	0.07 %	0.06 %	0.08 %	0.08 %	0.03 %

Table S4. Primers used to identify Isolated stains with MLST

Target gene	Primer name	Primer sequence (5′—3′)	Fragment length (bp)	Source or reference
16S rRNA	27F	AGAGTTTGATCCTGGCTCAG	1465	
	1492R	GGTTACCTTGTTACGACTT		
gyrB	PfgyrBF	TGCACGGYGTRGGYGT	939	(1)
	PfgyrBR	CMGCRGAGTCACCTTCCA		
rpoD	PsEG30F	ATYGAAATCGCCAARCG	763	(2)
	PfrpoD 804R	CCTCRCCGATCGACATG		(1)
rpoB	LAPS	TGGCCGAGAACCAGTTCCGCGT	1230	(3)
	LAPS27	CGGCTTCGTCCAGCTTGTTCAG		
racA	recAF126	NCAGATYGARAAGCAGTTTYGG	770	(4)
	recAR928	RCCGYYRTAGSYRTACCASGC		

M, A+C; R, A+G; Y, C+T; K, G+T.

20s at the annealing temperature for each gene (55˚C for 16S rRNA, 57˚C for gyrB, 54˚C for rpoD, 62˚C for rpoB and 58˚C for racA).

References:

1. **Tanaka, C., K. Yamada, H. Takeuchi, Y. Inokuchi, A. Kashiwagi, and T. Toba.** 2018. A Lytic Bacteriophage for Controlling Pseudomonas lactis in Raw Cow's Milk. APPLIED AND ENVIRONMENTAL MICROBIOLOGY **84**.

2. **Mulet, M., A. Bennasar, J. Lalucat, and E. García-Valdés.** 2009. An rpoD-based PCR procedure for the identification of Pseudomonas species and for their detection in environmental samples. Molecular and cellular probes **23:**140-7.

3. **Ait Tayeb, L., E. Ageron, F. Grimont, and P. A. D. Grimont.** 2005. Molecular phylogeny of the genus Pseudomonas based on rpoB sequences and application for the identification of isolates. Research in microbiology **156:**763-73.

4. **Gomila, M., C. Prince-Manzano, L. Svensson-Stadler, A. Busquets, M. Erhard, D. L. Martinez, J. Lalucat, and E. R. B. Moore.** 2014. Genotypic and Phenotypic Applications for the Differentiation and Species-Level Identification of Achromobacter for Clinical Diagnoses. PLOS ONE **9**.

Table S5. Results of ANIb data using the JSpeciesWS comparison with *P. GXZC* strain

RefSeq assembly accession	Genome	ANIb [%]	Aligned [%]	Aligned [bp]	Total [bp]
GCF_000503215.1	Pseudomonas canadensis 2-92 [T]	92.4	75.74	5346996	7059625
GCF_021605905.1	Pseudomonas canadensis PA-6-2A	92.24	74.78	5279051	7059625
GCF_900111895.1	Pseudomonas simiae CCUG 50988 [T]	90.55	72.05	5086351	7059625
GCF_000934565.1	Pseudomonas simiae PCL1751	90.54	72.3	5103965	7059625
GCF_900103345.1	Pseudomonas azotoformans LMG 21611 [T]	89.64	75.99	5364308	7059625
GCF_002007785.1	Pseudomonas azotoformans PF77.fasta	89.57	75.28	5314418	7059625
GCF_024112395.1	Pseudomonas pergaminensis 1008 [T]	89.53	75.19	5307889	7059625
GCF_013392005.1	Pseudomonas allii MAFF 301514 [T]	89.51	74.03	5226192	7059625
GCF_900104365.1	Pseudomonas extremorientalis LMG 19695 [T]	89.18	74.32	5246756	7059625
GCF_001645105.1	Pseudomonas marginalis ICMP 3553 [T]	88.84	71.12	5020893	7059625
GCF_021166635.1	Pseudomonas petroselini MAFF 311094 [T]	88.74	71.66	5058745	7059625
GCF_002563895.1	Pseudomonas lurida LMG 21995 [T]	88.74	72.06	5087208	7059625
GCF_014268275.3	Pseudomonas tritici SWRI145 [T]	88.48	70.07	4946640	7059625
GCF_014268375.2	Pseudomonas salmasensis SWRI126 [T]	85.68	64.24	4535161	7059625
GCF_902329575.1	Pseudomonas carnis [T]	85.68	64.41	4547276	7059625
GCF_904063055.1	Pseudomonas paracarnis V5/DAB/2/5 [T]	85.51	64.55	4556696	7059625
GCF_019145205.1	Pseudomonas khavaziana SWRI124 [T]	85.5	65.29	4609218	7059625
GCF_001439845.1	Pseudomonas lactis DSM 29167 [T]	85.48	67.05	4733285	7059625
GCF_001439735.1	Pseudomonas paralactis DSM 29164 [T]	85.44	64	4518075	7059625
GCF_900101035.1	Pseudomonas libanensis DSM 17149 [T]	85.42	65.36	4614155	7059625

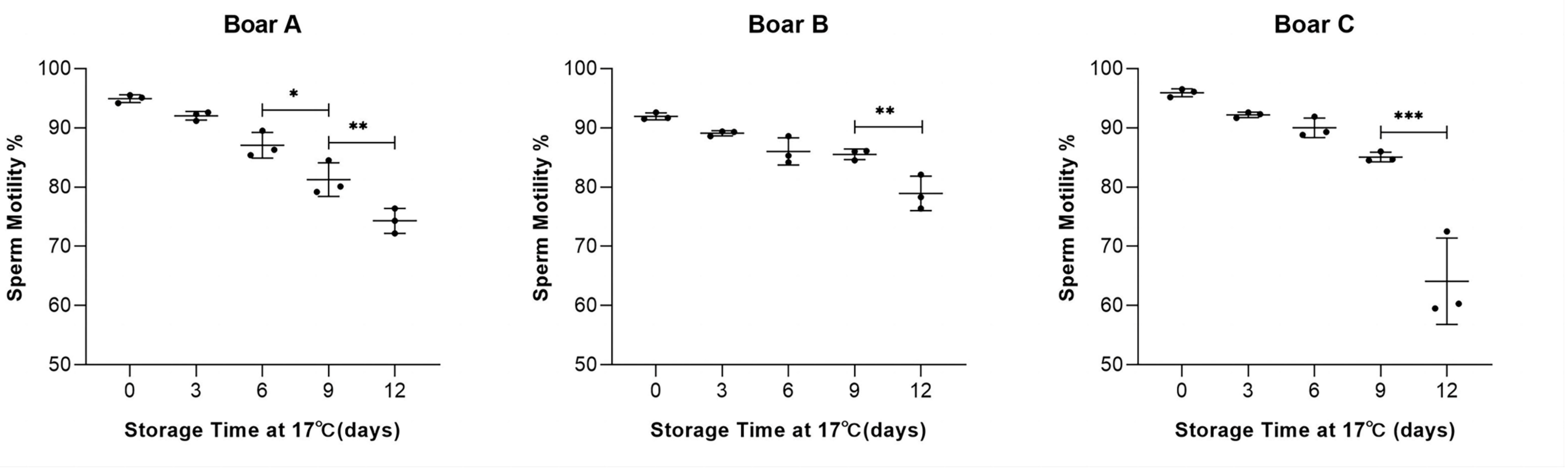

Figure S1. Total sperm motility of extended semen stored at 17 °C for days

Data are shown as mean ± SD, n = 3. *: $p < 0.05$, **: $p < 0.01$, ***: $p < 0.001$, "ns" was not shown.

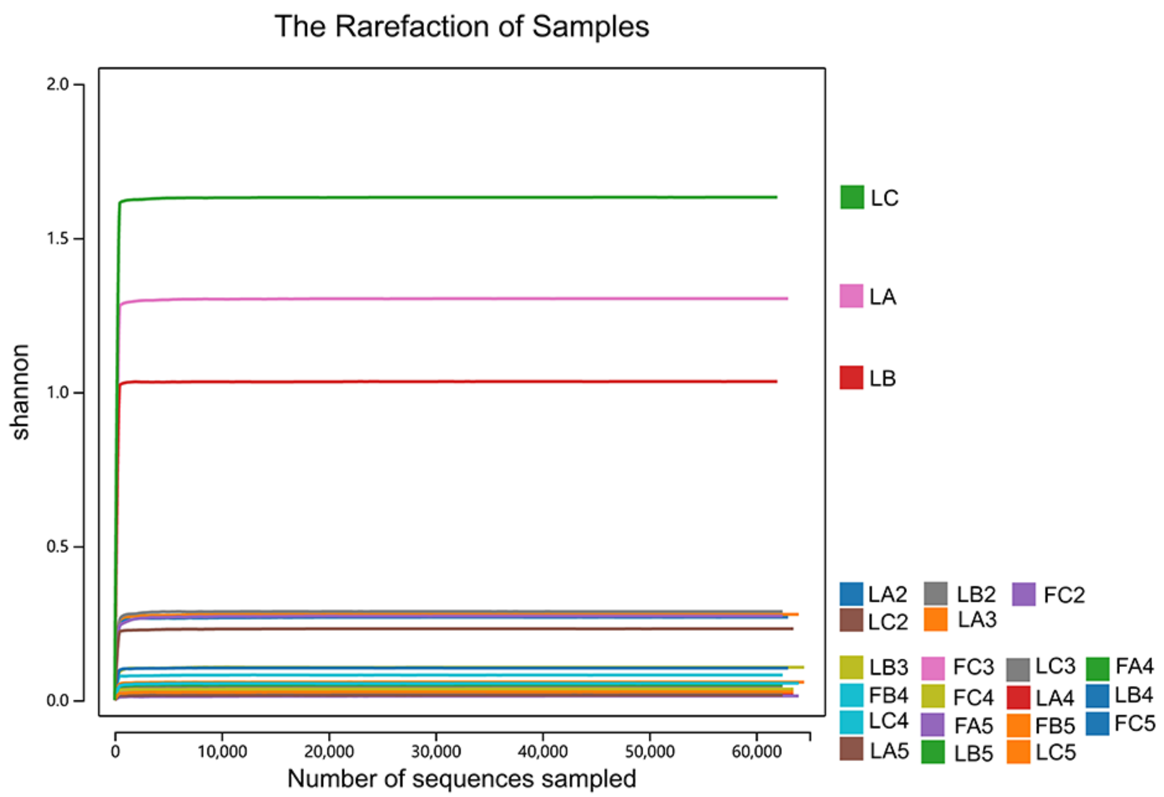

Figure S2 The Shannon diversity index of samples

The Shannon diversity index was calculated at genus level. The horizontal coordinate represents the amount of sequencing data and the vertical coordinate is the index of Shannon diversity. The higher index indicates higher diversity of microbial community(1).

References:

1. **Shannon, C.** 1997. The mathematical theory of communication (Reprinted). M D COMPUTING **14:**306-317.

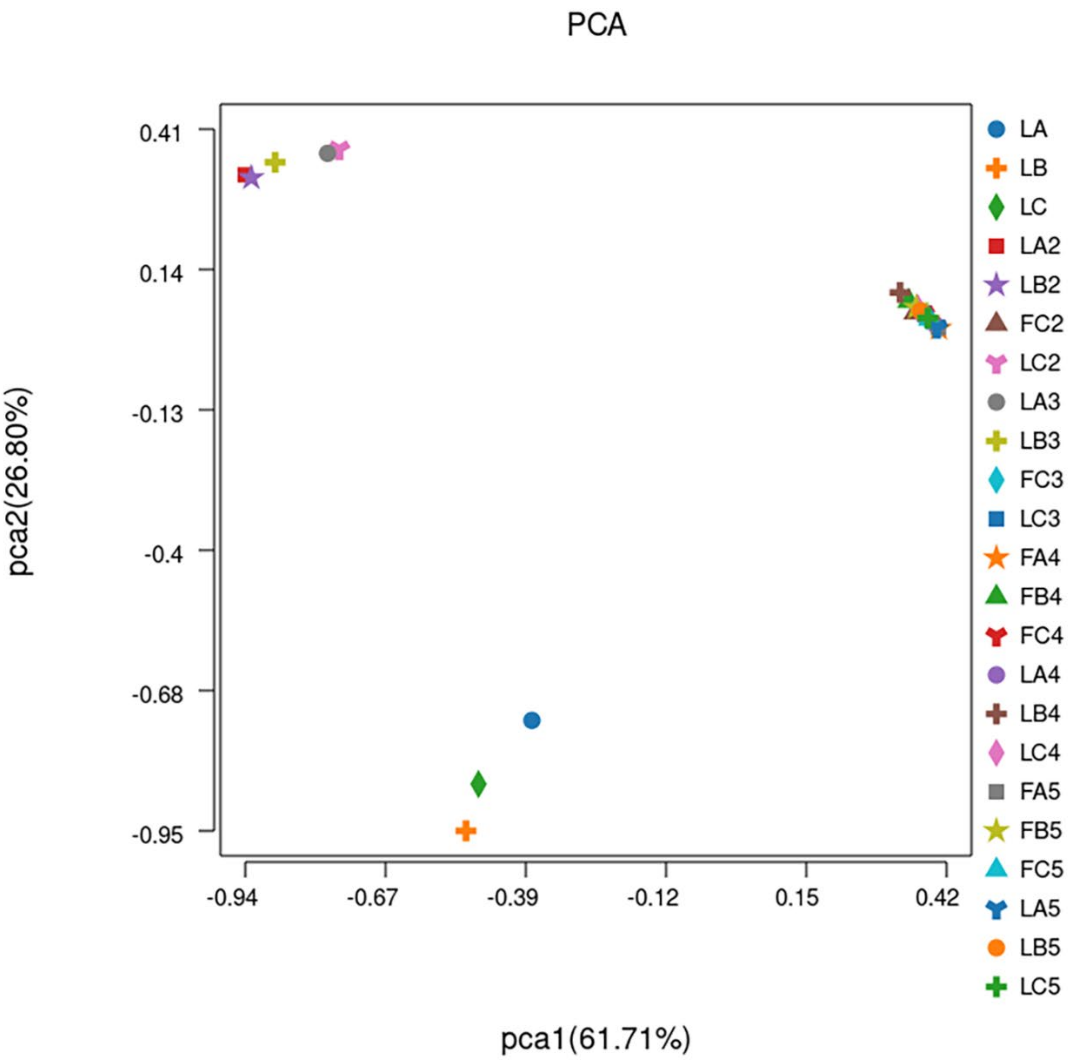

Figure S3 Principal Components Analysis (PCA)

Supplementary Methods

The commercial extender Zenolong® (Beikang, Taizhou, China), a long-term preservation extended, is widely used for semen dilution and preservation in the artificial insemination (AI) station with nearly 10,000 boars (Yangxiang Farming Co., Ltd).

As commercial extenders, the Zenolong® exact composition cannot be described, but the main components are same as Beltsville thawing solution (BTS) (information from suppliers), such as, glucose, sodium citrate, sodium bicarbonate, EDTA. The main difference between the two extenders is the variety of substances, including antibiotics, antioxidants, stabilizer, etc., which prolong the preservation of extended semen. To explore the bacterial composition of extended semen with absence of antibiotics, we set the extended semen samples diluted in BTS.
